# Obtaining Parallel Sentences in Low-Resource Language Pairs with Minimal Supervision

**DOI:** 10.1155/2022/5296946

**Published:** 2022-08-03

**Authors:** Xiayang Shi, Ping Yue, Xinyi Liu, Chun Xu, Lin Xu

**Affiliations:** ^1^College of Software Engineering, Zhengzhou University of Light Industry, Zhengzhou 450000, China; ^2^College of Mathematics and Information Science, Zhengzhou University of Light Industry, Zhengzhou 450000, China; ^3^Xinjiang University of Finance and Economics, Urumqi 830012, China; ^4^Control Engineering College, Chengdu University of Information Technology, Chengdu 610225, China; ^5^School of Intelligent Medicine, Chengdu University of Traditional Chinese Medicine, Chengdu 611137, China

## Abstract

Machine translation relies on parallel sentences, the number of which is an important factor affecting the performance of machine translation systems, especially in low-resource languages. Recent advances in learning cross-lingual word representations from nonparallel data by machine learning make a new possibility for obtaining bilingual sentences with minimal supervision in low-resource languages. In this paper, we introduce a novel methodology to obtain parallel sentences via only a small-size bilingual seed lexicon about hundreds of entries. We first obtain bilingual semantic by establishing cross-lingual mapping in monolingual languages via a seed lexicon. Then, we construct a deep learning classifier to extract bilingual parallel sentences. We demonstrate the effectiveness of our methodology by harvesting Uyghur-Chinese parallel sentences and constructing a machine translation system. The experiments indicate that our method can obtain large and high-accuracy bilingual parallel sentences in low-resource language pairs.

## 1. Introduction

In many languages natural language processing applications, the most widely used and most important data are parallel sentences, which play a more important role in statistical machine translation (SMT) and neural machine translation (NMT). Thus, many approaches have been achieved to obtain bilingual sentences from multilingual websites and achieved great success [1–4]. They can be divided into two ways: (i) The first category mainly uses multiple features such as tags in URLs, link anchors, image alt, HTML tags to identify parallel pages by calculating the similarity of features, thereby to identify whether web pages in two languages are mutually translated. (ii) The way others choose parallel sentences is achieved by constructing a classifier. These include maximum entropy classifiers, Bayesian neural networks, and support vector machines [5–14]. Both methods have proven prospects to get bilingual corpus in certain language pairs. However, these methods have limitations. They apply to certain specific websites or rich language pairs. Then, in the face of bilingual languages with insufficient resources, they still do not perform well in obtaining bilingual corpus.

Two major challenges are the difficulty to obtain bilingual parallel sentences in low-resource language pairs. First, dynamic news websites are created with modules and the contents are published in different languages. As a result, it is difficult to recognize two parallel pages by the URLs, the other features have the same problems such as HTML tags and images. For example, previous works obtained parallel language corpora from Wikipedia and Twitter [15–18]. Because the structure design of many news websites is very complicated now, it is difficult to obtain multiple features. Second, the classifier is a good solution. It can select parallel corpora from a large number of noisy data. To implement this process, we must have enough parallel data to train a good classifier. The number of parallel sentences has an impact on the classifier [19, 20]. For example, Francis Gregoire et al. use 60k parallel sentences to train a neural network classifier. However, training parallel sentences are invaluable in low-resource language pairs.

In this paper, we first induce bilingual signals by establishing continuous word embeddings for cross-language mapping. Then, using a word-overlap model obtains training parallel sentences to construct a classifier. Finally, we extract parallel sentences by constructing a long- and short-term memory bidirectional recurrent neural network (LSTM-BiRNN) classifier. In this process, our methodology can obtain parallel sentences via hundreds of bilingual words. For the proposed method, we use the Uyghur-Chinese language pair, and build an SMT system for experiments, and verify the effectiveness of the method through BLEU scores. By experiments have also shown that we can obtain exceptional results by eliminating the need for any particular feature works or external resources.

## 2. Related Works

The amount of information available for natural language processing applications on the Internet is rapidly expanding, and many methods try to grab network data to build a training corpus. At the same time, a variety of methods have been developed to extract parallel sentences. They can be roughly divided into two forms.

First, many approaches use content-based metrics to select parallel sentences [21–25], such as bag-of-words overlapping, SVM classifier, and neural network classifier. These methods have been proven. Although they are useful for selecting parallel data, they have certain limitations. They require sufficient language resources (such as bilingual dictionaries, parallel sentences, or basic machine translation systems). Some low-resource language pairs may not be able to obtain data. For example, [9] proposed the use of a twin bidirectional recurrent neural network (BiRNN) to construct the most advanced classifier, and use the classifier to detect parallel sentences. This method eliminates the dependence on feature engineering in any specific domain and avoids relying on multiple models and original parallel sentences. However, parallel sentences are still very valuable in resource-poor language pairs. Therefore, this method may not be suitable for languages with relatively scarce resources [26].

In addition, many other people's work uses features such as HTML structure, URL, and image alt of web pages to detect possible parallel sentences [22, 27–30]. For example, there are links between translated articles in Wikipedia, and Smith et al. (2010) [5] use these links to grab parallel sentences or words. These methods are very useful in some specific websites and have been proven. The biggest challenge is to extend these methods to unsupervised web crawling strategies.

Esplà et al. (2016) [29] built a tool called Bitextor, which is an open source and free for users to use. It collects parallel data from multilingual websites. It adopts a highly modular design, to allow users to easily obtain parallel corpora from the Internet, and the obtained corpus is segmented and aligned. It uses a bilingual dictionary to compare the HTML structure of the document and the number of aligned words to obtain parallel sentences. According to the bilingual dictionary provided by the user, the system can automatically and quickly compare parallel data. But for those language pairs with insufficient resources, it is difficult to obtain the desired bilingual dictionary, which is also the biggest challenge.

## 3. Methodology

**Figure 1:**
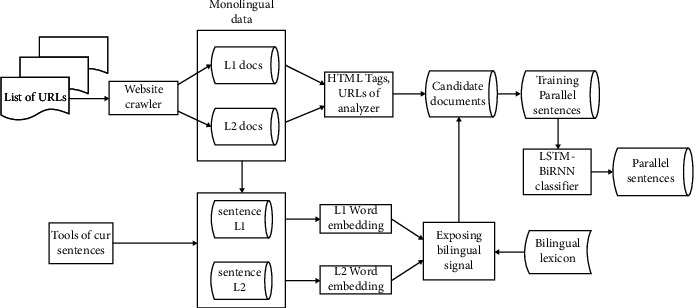
The architecture of obtaining parallel sentences.

In this section, we will explain how to obtain the source of data in detail [1] and how to induce the final parallel corpus. The data source determines the parallel corpus to be obtained. Use web crawler technology to obtain monolingual data and then construct the obtained data into continuous word representations. Then, we construct the cross-lingual word representation mappings to induce bilingual signals which inspired by [31]. With bilingual signal and constructing classifier, we can induce parallel sentences. The general architecture of our obtaining parallel corpus is presented (see Figure 1).

### 3.1. Crawling Web Data and Candidate Documents

**Figure 2:**
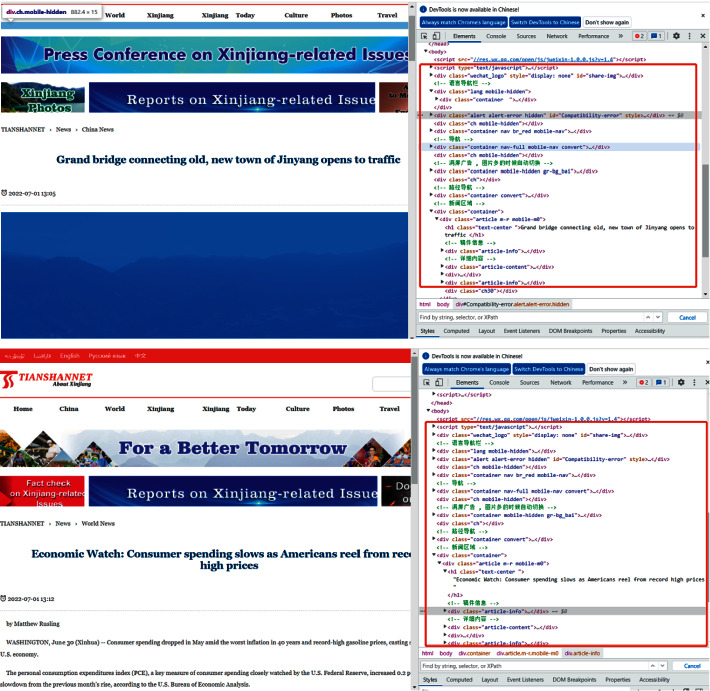
Two different contents of web pages have the same structure.

Like most approaches, the first step downloads the whole website. We download the body of news pages. As the current website adopts a highly modular design, usually when the themes of the pages are the same, their HTML structure is mostly the same (see Figure 2).

Therefore, if only comparing HTML tags structure, we can find that the two pages are the same. However, this is not the case. Scrapy toolkit is a very convenient toolkit. Users can grab some parts of web pages. We use this tool to grab web page data. The second step is the selection of candidate document pairs. A website may contain tens of thousands of documents. If we do not filter, the matching process is very inefficient and the results are very inaccurate. To solve this problem, we add a time window based on the idea of [6]. According to the timeliness of news sites, check the release time of each page (see Figure 2).

The same topic multilingual documents often are reported in the same period. Thus, we use the following heuristic: We assume that two news items with similar content may have similar release dates. Therefore, for each query, we only query the news data within a few days before and after the release date of the target document. According to the above query process, we set the time window size to three days. Then, in order to obtain higher accuracy, each query can only search for fewer documents. We will introduce how to recognize that two multilingual documents are parallel, in the next section.

### 3.2. Inducing Bilingual Signal

In this paper, similar to most approaches that induce bilingual lexicon from nonparallel data, the usual task that learns a high-coverage bilingual lexicon, our objective is harvesting a precise bilingual signal from monolingual data. Our objective function:

(1) $$\begin{array}{c} J\left(w_{v^{s}}^{i},w_{v^{t}}^{j}\right)=\min\left(\alpha J_{\text{mono}}+\left(1-\alpha \right)J_{\text{match}}\right), \end{array}$$

where *w*_*v*^*s*^_^*i*^ is the one word in the vocabulary of *v*^*s*^, while the reverse direction follows by symmetry for *w*_*v*^*t*^_^*j*^. *J* is the score of two words that have similar semantics. *J*_mono_ mainly explain the similar semantic score that a word is from a seed word.

Unlike the usual monolingual term *J*_mono_ explaining regularities in monolingual corpora, we explain the mutual translation probability of two words in terms. When two words have similar meanings, their word distances are closer. Then we can measure the distance between the two words and the seed, and reveal more translation pairs through the distance. Assuming that these two words are closer to the seed, the two words have a greater probability of translating each other:

(2) $$\begin{array}{c} J_{\text{mono}}=J_{\text{mono}}^{s}+J_{\text{mono}}^{t}, \end{array}$$

(3) $$\begin{array}{c} J_{\text{mono}}^{s}=\underset{ss\in D}{\min\;\cos}\left(w_{{}^{v^{s}}}^{i},w_{{}^{v^{s}}}^{ss}\right), \end{array}$$

(4) $$\begin{array}{c} J_{\text{mono}}^{t}=\underset{tt\in D}{\min\;\cos}\left(w_{v^{t}}^{j},w_{v^{t}}^{tt}\right). \end{array}$$

Our monolingual term *J*_mono_^*s*^ encourages the source embeddings of word translation pairs in a seed lexicon *D* to move closer. *w*_*v*^*s*^_^*ss*^ is the ss-th word in a seed lexicon D. ss and tt are a pair words of D. For the detailed calculation of *J*_mono_^*s*^, we use the cosine function. For the target, we use the same process.

The *J*_match_ object can show how target words from a source have been translated into another. When we learn the bilingual signal from the same language corpus, it means that the source word vector and the target word vector are independent training. The two parts are not in the same position. To solve this problem, matching items can be induction:

(5) $$\begin{array}{c} J_{\text{match}}=\underset{\left(ss,tt\right)\in D}{\min}\left\|J_{\text{mono}}^{s}-J_{\text{mono}}^{t}\right\|. \end{array}$$

Using a simple example to explain this procedure, assume that we have an English lexicon {perform, believe, talk} and a Chinese lexicon {zhixing, shishi, jiaotan}, an English–Chinese bilingual lexicon {conduct: jinxing}. Assuming that it has already conducted the formulas (3) and (4), we can calculate that {perform} is closer with {conduct} in source and {zhixing, shishi} are closer with {jinxing} in the target. Then, we further calculate formula (5) and we can add {perform: zhixing} into the original lexicon.

### 3.3. Obtaining Parallel Sentences

For our particular situation that is seriously low-resource language pairs, although classifier is a good method to identify parallel sentences, we do have not enough parallel sentences to train this classifier. In the initial stage, we use a word-overlap model filter to select parallel sentences.

The word-overlap model must rely on bilingual dictionaries, and parallel sentences can be identified by the number of cooccurring word pairs. This process can be expressed as follows:

(6) $$\begin{array}{c} \text{score}\left(L_{s},L_{t}\right)=\frac{\sum L_{s}\cap L_{t}}{\max\left(\text{len}\left(L_{s}\right),\text{len}\left(l_{t}\right)\right)}. \end{array}$$

We use ∑*L*_*s*_∩*L*_*t*_ to count the number of translation words of the source sentence and the target sentence. With the above content, we can conclude that the most important step in this method is to induce bilingual signals. To quickly calculate the alignment of the source sentence and the target sentence, we can use a bilingual dictionary to achieve. Although, we may not get a bilingual dictionary with a large coverage. As a result, using the word-overlap model, we can get several parallel sentences. We can also see this in the experiment.

**Figure 3:**
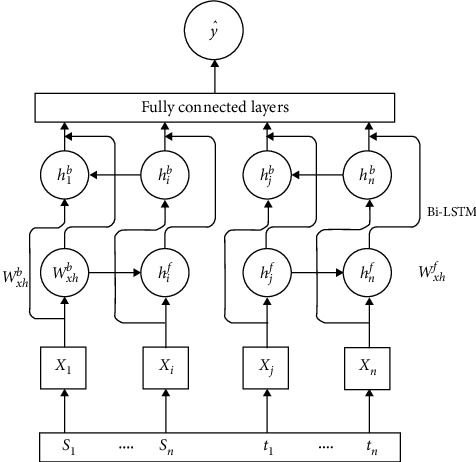
Architecture for bidirectional recurrent neural networks. The fully connected layers predict the probability that two sentences are translated to each other.

In order to get more parallel sentences, we further use a good classifier to get more parallel data. On the one hand, recent advances in deep neural networks have shown that they can successfully learn complex mapping from variable-length sequences to continuous vector representations. On the other hand, deep neural networks do not rely on a significant amount of feature engineering. In this paper, we construct a bidirectional recurrent neural network (Bi-RNN) classifier to filter parallel sentences. Our neural network architecture is illustrated in Figure 3 (see Figure 3).

In the past, most of them used the method of transforming parallel sentences into vectors to train the classifier of the neural network, and we also used the same method. However, instead of directly using the word vector as input, we use a fixed sentence vector size as the input strategy. For the input of the *X* layer, we define it as follows:

(7) $$\begin{array}{c} x_{i}=\text{sigm}\left(w_{i}V_{i}+b\right). \end{array}$$

The Bi-RNN layer contains a feed-forward and feeds backward neural networks layer. This can be described by the following equation:

(8) $$\begin{array}{c} h_{i}^{f}=\phi \left(w_{xh}^{f}\cdot x_{i}+w_{hh}^{f}\cdot h_{i-1}+b^{f}\right),\\ h_{i}^{b}=\phi \left(w_{xh}^{b}\cdot x_{i}+w_{hh}^{b}\cdot h_{i-1}+b^{b}\right),\\ h=h_{i}^{f}⊙h_{i}^{b}. \end{array}$$

For the prediction part, we set a threshold. When the probability value exceeds the threshold, the sentence is recognized as parallel. We can calculate as follows:

(9) $$\begin{array}{c} \hat{y}=\left\{\begin{array}{cc} 1, & \text{if }p\left(y=1|h\right)>\sigma ,\\ 0, & \text{otherwise}. \end{array}\right.. \end{array}$$

## 4. Experiments

We evaluate the effectiveness of our method by running baselines in different environments. Because our main purpose is to solve the problem of insufficient resources in the process of multilanguage processing. We conducted a detailed study of low-resource language pairs and used them as an example of a target.

### 4.1. Experiment Setup

Evaluations and Ground Truth: In order to objectively evaluate the parallel sentences we obtained, we adopted two methods to do. The first is the accuracy rate, that is, the proportion of truly parallel sentence pairs in all obtained sentence pairs. When our data are obtained through an open platform, we have no professional translators manually annotate parallel sentences from the open dataset. Thus, to carry out the evaluation, we use the CWMT′17 2 Uyghur to Chinese parallel dataset. In the actual evaluation, we first randomly add the sentences of the CWMT′17 dataset into the open dataset. Then, we select the source sentences of obtained parallel sentence pairs that appear in the CWMT′17 dataset and compute the accuracy by retrieve target sentences whether exist in the CWMT′17 dataset.

The other is to use a machine translation system to translate the obtained parallel sentences and use the BLEU score as an evaluation indicator.

Data: In this experiment, the experimental system obtains bilingual parallel sentences through three multilingual news websites: TianShan^3^, RenMin^4^, and KunLun^5^. We select web pages with more than 20 words in the document content. The statistical results of the preprocessed corpus are shown in Table 1. We choose sentences longer than 10 words.

Baseline: For experimental comparison, we use the Bitextor parallel sentence extraction system developed by Esplà-Gomis et al. The system is free and open source for users, and it is used to obtain parallel data from multilingual websites. When in use, the user provides one or more website addresses that need to be processed to the system, and the system can automatically download multilingual data. Moreover, a big bilingual lexicon is required in the process. Then, it can automatically analyze the structure of web pages and obtain parallel data. Therefore, we conduct experiments by setting bilingual dictionaries of different sizes to test how the size of bilingual dictionaries affects the acquisition of parallel sentences.

Another problem is to evaluate the classifier. We all know that training classifiers must use parallel sentences, and use the classifier to predict parallel sentences. The size of parallel sentences will also affect the performance of the classifier. So we select different numbers of parallel sentences to train the classifier and test it.

### 4.2. Overall Performance

In order to study the effectiveness of using our system to obtain parallel sentences in low-resource language pairs, we run our system and the Bitextor system separately, and the experimental environment is set in low-resource language pairs. Due to the timeliness of news, our system uses time windows to select parallel data, but the Bitextor system does not use this feature to extract data. In order to ensure the consistency of the experiment, we use a time window to filter the results of Bitextor.

Table 2 shows the performance of our method and the Bitextor in low-resource language pair. For computing accuracy, we follow the evaluation in Experiment. We only select the sentences that appear in CWMT′17 to compute the accuracy. Compared to our method, the baseline attains considerably lower performance. Where No means that features are not used to obtain candidate sentence pairs, URL means that candidate sentence pairs are obtained by URL filtering, and URL + HTML means that both URL and HTML tags are used to obtain candidate sentence pairs. It can be seen from the table that the accuracy of obtaining candidate bilingual sentence pairs based on multiple features is the highest. The poor performance should be attributed to the harsh condition that we have only 600 seed words. The Bitextor needs enough bilingual lexicon to ensure this system can obtain high precision bilingual sentences. However, the success of our method demonstrates that it is possible to obtain parallel sentences in low-resource language pairs.

As shown in the table, the effect of extracting parallel statements by our system is much higher than that of bitextor. When no filtering feature is used to extract candidate corpus, the accuracy of our system is 0.67, which is higher than that of bitextor 0.55. In the case of the same system, when adding URL and HTML feature filtering methods, the accuracy of bitextor system is 0.11 higher than that without adding feature filtering methods, and our system is 0.18 higher than that without adding feature filtering methods. It shows that our system and the method of obtaining corpus through feature filtering proposed in this paper are feasible.

Our method makes full use of the limited bilingual lexicon to induce more translated words and use an excellent classifier to obtain parallel sentences. Our method does not use the URLs et al. as the measure to select parallel sentences. The Bitextor also obtains lots of candidate parallel sentences, but the precision is very low. We attribute that it uses multifeatures such as HTML tags as the measure to select parallel sentences. In the above section, we already analyze the shortage of using multifeatures.

### 4.3. Effect of Bilingual Lexicon Size

**Figure 4:**
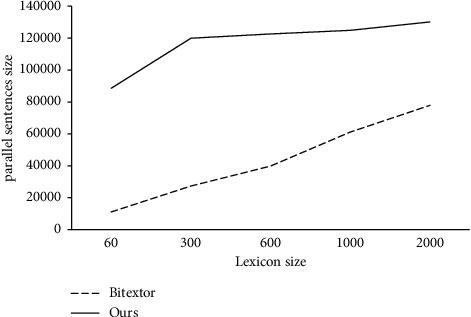
Size of the result as the entries of the bilingual lexicon.

**Figure 5:**
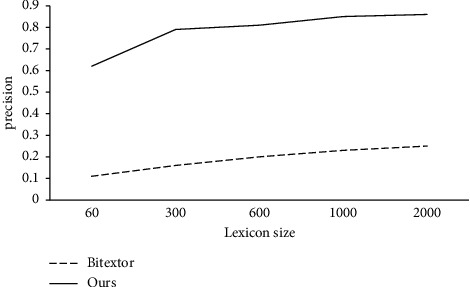
Accuracy of the result as the entries of the bilingual lexicon.

During the training process, we view the obtained results by changing the dictionary size, and record the performance, as shown in Figures 4 and 5.

In the experiment, we used {60; 300; 600; 1,000; 2,000} entries to obtain parallel sentences. From Figures 4 and 5, we can see that bilingual dictionaries have a great influence on the process of obtaining parallel sentences. We can see from the figure that as the size of the bilingual dictionary becomes smaller, it becomes very difficult for Bitextor to obtain parallel sentences. However, we can see that our system has achieved very good results with low resources.

With the increasing of lexicon entry, Bitextor can gradually obtain more and more parallel sentences and the accuracy is also higher. However, ours still stay relatively stable results. In the experiment, when the number of dictionaries is set to 60, the number and accuracy of the parallel sentences we obtain are relatively good. Combined with the actual performance of the baseline, this result is in line with our expectation of obtaining parallel sentences in low-resource language pairs.

### 4.4. Effect of Parallel Sentences for Classifier Experiments

In this section, we will construct a bilingual classifier to extract bilingual parallel sentences. Moreover, we discuss the classifier how affects the obtaining parallel sentences, and which factors affect it.

In this experiment, we constructed three classifiers: LSTM bi-directional recurrent neural network (LSTM-BiRNN) classifier, simple three-layer recurrent neural network (RNN) classifier, and support vector machine (SVM). At the same time, we use 2000; 5,000; 10,000; 20,000; 40000 parallel sentence training classifiers.

Table 3 shows the results of the Uyghur-Chinese experiment. We can observe that the three classifiers show different accuracy and scale. It can be seen from the above table that LSTM-BiRNN shows better results than other networks in the experiment. Therefore, we believe that more and better usage information can be obtained through the LSTM-BiRNN mechanism. However, support vector machine has the disadvantages of difficult training for large-scale data, sensitive to missing data and high computational complexity, which leads to the worst extraction effect. Another interesting finding is that when the number of training parallel sentences is set to 2000, the three test systems can only get fewer parallel sentences, and the accuracy of sentences is also very low. Therefore, the size of the training corpus also has a great impact on the acquisition of parallel sentences. However, we have gradually increased the number of training parallel sentences. No matter RNN, LSTM-BiRNN, or SVM, we can see a great improvement in scale and accuracy. When the corpus is up to 40000, LSTM-BiRNN has the best effect in extracting parallel sentences, with an accuracy of 0.85, while SVM has the worst effect, with an accuracy of 0.69. From the above results, we can see that the number of training parallel sentences is an important reason that affects the performance of the classifier. It also shows that inducing bilingual signals is very important for us. We elaborate on this approach. Only by using this method to obtain enough parallel sentences can the best classifier be trained.

### 4.5. Machine Translation Evaluation

We obtain parallel sentences through the system, and the goal of obtaining data is to build a translation system used in low-resource sentence pairs. In order to prove the effectiveness of our method, we build a low-resource Uyghur–Chinese language pair machine translation system, and use the obtained parallel sentences to test, and evaluate its quality through experimental BLEU scores. We use phrase-based translation system training, evaluated with open source and free Moses [32].

We obtain the respective parallel sentences through Bitextor and our system, respectively, in order to provide data for the translation system for the low-resource parallel sentences we constructed. The reason for using Bitextor is that we need a baseline system to measure. For two methods, we use {1,000; 2,000} seed lexicon entries to obtain parallel corpus to train the machine translation system. The first experiment is to obtain enough parallel sentences to provide a data set for machine translation (Table 4). Where No-Ours stands for the dictionary created using mapping, and Ours stands for the dictionary created using mapping method. We can see that under the same conditions, although the two systems have obtained parallel sentences, our system extraction results are obviously due to bitextor, and the result of extracting bilingual parallel sentence pairs after adding the mapped dictionary is the best, with an accuracy of 0.85. Through analysis, we believe that this is because bitextor needs enough bilingual dictionaries. It can also be seen from the table that the larger the dictionary, the better the extraction effect of bilingual parallel sentence pairs. Next, we use the parallel sentences obtained when training the Uyghur–Chinese machine translation system.  Table 5 shows the BLEU scores.

Through experimental comparison, we can see that our method has obtained a higher BLEU score than the baseline. In Tables 4 and 5, we think that the SMT system has low performance because the baseline system cannot obtain a higher BLEU score. A parallel corpus of accuracy. We all know that the quality of the training corpus is one of the most important factors affecting the performance of the SMT system. Through further analysis, we know that if Bitextor wants to obtain a higher-precision parallel corpus, it needs to provide a bilingual dictionary with larger coverage. Although a parallel corpus can be obtained through Bitextor, Bitextor performs relatively poorly for low-resource language pairs. The analysis of the experimental results can clearly show that using our method, parallel sentences can be obtained in low-resource language pairs and have excellent performance. It should be noted that we can obtain low-resource language pairs through the above methods and build a low-resource machine translation system.

## 5. Conclusions

This paper presents a new method to obtain parallel sentences by minimum supervision. The main purpose of this method is to solve the problem of insufficient low-resource language corpora. Compared with the traditional system, our method shows better results in acquiring parallel corpora on multilingual websites, especially in low-resource language pairs. Our proposal consists of three steps. In the first step, we train the two monolinguals into continuous word representations. The second step is to use the word-overlap model to find parallel training sentences and provide data for the classifier. Finally, we construct an LSTM-BiRNN classifier to obtain more parallel sentences. In order to measure our method, we build an SMT system, and use the parallel statements obtained by our method to provide a data set for the experiment. Through experiments, it can be found that our method can obtain the most advanced results in low-resource language pairs through hundreds of bilingual entries.

## Figures and Tables

**Table 1:** Experiment set statistics.

Websites	Languages	#Wenpages	# Sentences
TianShan	Chinese	249,238	3,839,000
	Uyghur	48,907	427,000

RenMin	Chinese	451,972	5,500,000
	Uyghur	99,578	590,000

KunLun	Chinese	44,046	641,000
	Uyghur	27,419	324,000

CWMT′17	Chinese	-	150,000
	Uyghur	-	150,000

**Table 2:** The precision and size of obtaining parallel sentences with 600 seeds.

Model	Languages	Features	Sentences size	Accuracy
Bitextor	Chinese - Uyghur	No	31,431	0.12
		URL	37,593	0.19
		URL + HTML	43,162	0.23

Our	Chinese - Uyghur	No	99,846	0.67
		URL	113,628	0.78
		URL + HTML	122,875	0.85

**Table 3:** The size and accuracy of obtained parallel sentences in different sizes of training corpus.

Model		2,000	5,000	10,000	20,000	40,000
LSTM-BiRNN	Size	13,213	65,834	65,834	92,843	126,875
	Accuracy	0.6	0.73	0.80	0.83	0.85

RNN	Size	14,156	58,783	58,968	86,638	121,386
	Accuracy	0.48	0.61	0.69	0.72	0.75

SVM	Size	11,329	49,361	51,548	69,823	88,942
	Accuracy	0.37	0.55	0.57	0.63	0.69

**Table 4:** Statistics of the size and precision of parallel sentences.

Model	Lexicon size	Sentences	Precision
Bitextor	1,000	123,868	0.21
	2,000	151,162	0.23
No-ours	1,000	215,267	0.76
	2,000	219,936	0.79
Ours	1,000	240,368	0.84
	2,000	246,875	0.85

**Table 5:** Blue scores on Uughur–Chinese SMT using different training corpus.

Model	Lexicon size	Sentences	Blue
Bitextor	1,000	123,868	7.3
	2,000	151,162	7.6
Ours	1,000	240,368	29.14
	2,000	246,875	30.65

## Data Availability

The Coda and Corpus data used to support the findings of this study are available from the corresponding author upon request.
